# Preparation of Graphene Quantum Dots by Visible-Fenton Reaction and Ultrasensitive Label-Free Immunosensor for Detecting Lipovitellin of Paralichthys Olivaceus

**DOI:** 10.3390/bios12040246

**Published:** 2022-04-15

**Authors:** Ailing Yang, Yue Su, Zhenzhong Zhang, Huaidong Wang, Chong Qi, Shaoguo Ru, Jun Wang

**Affiliations:** 1 College of Physics & Optoelectronic Engineering, Ocean University of China, Qingdao 266100, China; 21170211095@stu.ouc.edu.cn (Y.S.); wanghuaidong@stu.ouc.edu.cn (H.W.); qichong@stu.ouc.edu.cn (C.Q.); 2 College of Marine Life Sciences, Ocean University of China, Qingdao 266003, China; zhangzhenzhong5391@stu.ouc.edu.cn (Z.Z.); rusg@ouc.edu.cn (S.R.)

**Keywords:** graphene quantum dots, visible-Fenton reaction, fluorescence resonance energy transfer, immunosensor, environmental estrogen, lipovitellin

## Abstract

The increasing levels of environmental estrogens are causing negative effects on water, soil, wildlife, and human beings; label-free immunosensors with high specificities and sensitivities are being developed to test estrogeneous chemicals in complex environmental conditions. For the first time, highly fluorescent graphene quantum dots (GQDs) were prepared using a visible-Fenton catalysis reaction with graphene oxide (GO) as a precursor. Different microscopy and spectroscopy techniques were employed to characterize the physical and chemical properties of the GQDs. Based on the fluorescence resonance energy transfer (FRET) between amino-functionalized GQDs conjugated with anti-lipovitellin monoclonal antibodies (Anti-Lv-mAb) and reduced graphene oxide (rGO), an ultrasensitive fluorescent “ON-OFF” label-free immunosensor for the detection of lipovitellin (Lv), a sensitive biomarker derived from Paralichthys olivaceus for environmental estrogen, has been established. The immunosensor has a wide linear test range (0.001–1500 ng/mL), a lower limit of detection (LOD, 0.9 pg/mL), excellent sensitivity (26,407.8 CPS/(ng/mL)), and high selectivity and reproducibility for Lv quantification. The results demonstrated that the visible-Fenton is a simple, mild, green, efficient, and general approach to fabricating GQDs, and the fluorescent “ON-OFF” immunosensor is an easy-to-use, time-saving, ultrasensitive, and accurate detection method for weak estrogenic activity.

## 1. Introduction

Graphene quantum dots (GQDs) are a type of fluorescent carbon nanodot with a lateral size of less than 10 nm consisting of 3–10 layers of graphene (Gr) at the core and oxygen-containing chemical groups at the edge [1,2]. GQDs exhibit a strong quantum confinement and edge effect, resulting in extraordinary physical, chemical, electronic, and biological properties, which enable various significant applications in fluorescent imaging [3,4], optoelectronic devices [5,6,7,8,9], sensing [10,11,12], photovoltaic [13,14], photocatalysis [15,16], biomedicine [3,17], diagnosis, and therapeutics [4,18]. The excellent photo-physical properties, chemical inertness, water solubility, biocompatibility, low toxicity, and easy modification render GQDs as a new-generation luminescent nanomaterial. Many techniques have been developed for the fabrication of GQDs, which can be generally classified into top-down [5,6,7,13,15,16,19,20,21,22,23,24,25] and bottom-up approaches [10,12,14,15]. It is reported that the Fenton reaction (photo-, electrochemical-, and sono-Fenton, et al.) is an effective and simple technique to obtain GQDs [25,26,27,28,29,30,31,32]. Under ultraviolet (UV) light irradiation, the photo-Fenton reaction of GO was initiated at the carbon atoms connected with the oxygen-containing groups [26,27,28]. With the help of a Fenton reagent (Fe^2+^/Fe^3+^/H_2_O_2_), the reaction of the GO sheets was accelerated, and, as a result, the GQDs could be generated on a mass scale [26,27,28]. In the UV-Fenton reaction, a high-power UV light source had to be used [26,27,28], so a great deal of heat was produced in the experiment. To control the reaction temperature, a water-cooling setup had to be used, which increased the experiment cost and caused energy waste. It was reported that visible-Fenton or solar-Fenton could be employed to degrade organic pollutants [33,34,35] without a water-cooling setup. Thus, the visible-Fenton reaction is a viable alternative for the fabrication of GQDs. Compared to the UV-Fenton, the experimental setup for the visible-Fenton reaction is simple and low-cost. As far as we know, there are no reports about using the visible-Fenton reaction to prepare GQDs.

With the rapid development of modern industries and wide use of agriculture and environmental estrogens, such as insecticides, plasticizers, polychlorinated biphenyls (PCBs), etc., these chemicals have entered into lakes, rivers, seas, and soil, and have caused environmental pollution. Via vegetables, fruits, meat, and water, estrogens can accumulate in the human body and ultimately threaten human health. For example, disrupting the endocrine system, abnormal gonad development, skewed sex ratios, and other undesirable phenomena [36,37]. So, the detection of environmental estrogens has attracted wide-spread attention. The Organization for Economic Cooperation and Development (OECD) has recommended the egg yolk protein, vitellogenin (Vtg), of water vertebrate and invertebrate animals as one of the biomarkers for endocrine disrupting [38]. These techniques with wide detection ranges, low LODs, fast measuring speeds, and high specificities and sensitivities are very important for sensors in environmental estrogen testing. Immunosensors have intrinsically high specificities because the reactions between the antigens and antibodies are highly specific. For monoclonal antibody-based immunosensors, a certain antibody can specifically identify an antigen among many matters [38], and thus immunosensing techniques are the developing direction for complex environmental applications [39,40,41]. In previous years, Vtgs were tested using conventional immunoassay techniques such as enzyme-linked immunosorbent assays (ELISAs) [42,43], radioimmunoassay (RIAs) [44], and Western blots [45]. Although these methods showed high specificities and sensitivities, enzymes [42,43], radioactive elements [44], or second antibodies [45] had to be used. The overall procedures were time-consuming and not suitable for large amounts of detection in a short time. In recent years, electrochemical [46,47] and optical sensors [48,49,50,51] have also been used to test Vtg. The biosensor based on impedance spectroscopy indicated a wide detection range (1000–8000 ng/mL), but a low sensitivity (420 ng/mL) [46]; the amperometry method on screen-printed arrays presented a low LOD (0.09 ng/mL) and a narrow linear range (0.25–7.8 ng/mL) [47]; the technique that employed optical waveguide lightmode spectroscopy showed a wide detection range (100–10,000 ng/mL) and a low testing limit (0.1 ng/mL) [48]; the surface-enhanced Raman scattering (SERS) technique exhibited the lowest LOD (5 pg/mL), but had a very narrow linear range (~0.2 ng/mL) [49]. Recently, immunosensors based on fluorescence resonance energy transfer (FRET) between GQDs and Gr or GO were developed to test DNA [52], IgG [53], and myocardial infarction [54]. As far as we know, there are no reports using this strategy to test Vtg or lipovitellin (Lv).

**Scheme 1:**
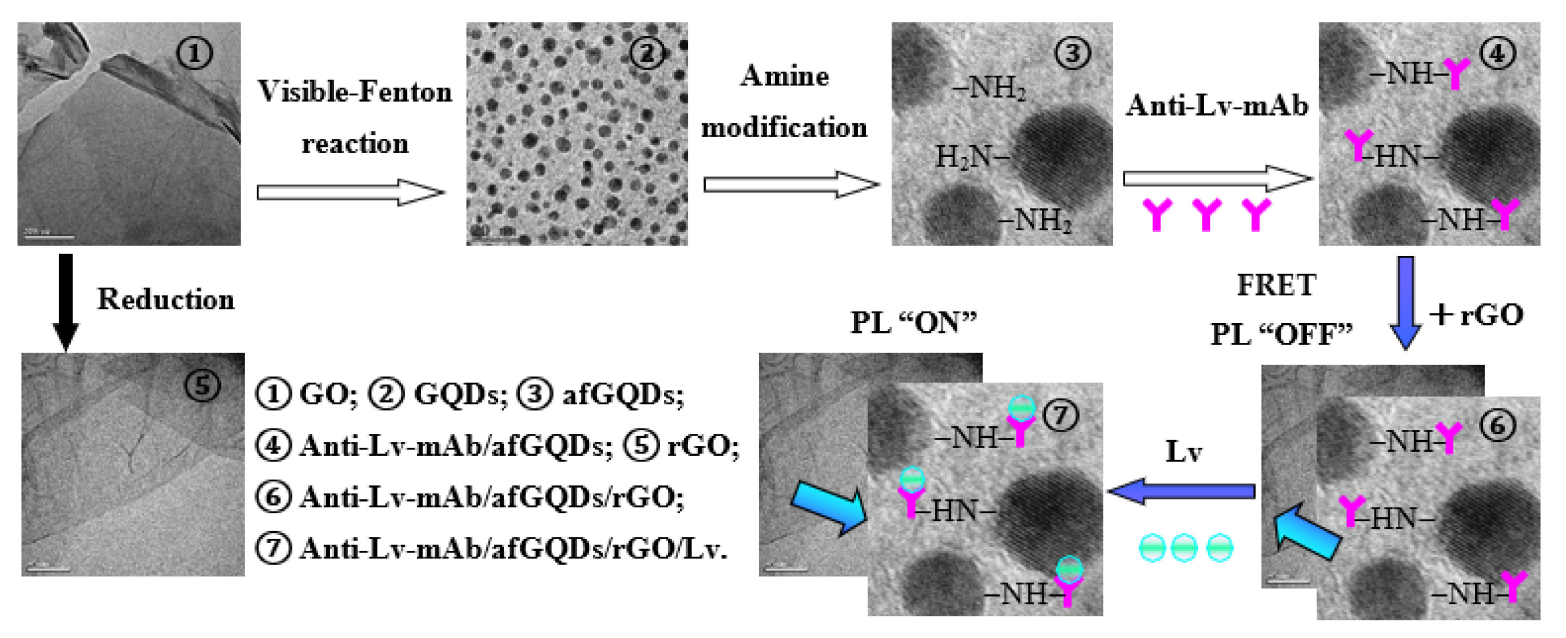
Schematic diagram of the immunosensor for detecting lipovitellin.

Inspired by this research and our previous study results, Lv, the main egg yolk protein derived from Vtg, was not only very stable, but also had the same binding efficiency to Anti-Lv-mAb as Vtg [55]. We established an ultrasensitive label-free “ON-OFF” immunosensor for detecting the Lv of Paralichthys olivaceus. The experimental procedure is in Scheme 1. With graphene oxide (GO) as a precursor, blue fluorescent GQDs were fabricated by using the visible-Fenton reaction. Furthermore, the morphology, size, structure, surface chemistry, and photo-physical properties of the GQDs were well characterized using a transmission electron microscope (TEM), high-resolution transmission electron microscope (HRTEM), atomic force microscope (AFM), X-ray diffractometer (XRD), Raman/Fourier transform infrared (FTIR), X-ray photoelectron spectrometer (XPS), and various other optical spectroscopy techniques. Using the amine-functionalized GQDs (afGQDs) conjugated to Anti-Lv-mAb as a fluorescent probe (afGQDs-Anti-Lv-mAb), an immunosensor for detection of the Lv of Paralichthys olivaceus was developed based on the FRET strategy, in which reduced GO (rGO) was used as an acceptor (quencher) and afGQDs-Anti-Lv-mAb as a donor. The quenched fluorescence could be recovered upon the addition of the target antigen Lv into the Anti-Lv-mAb/afGQDs/rGO (conjugate/rGO) solution. The fluorescence recovery degree is proportional to the concentration of Lv, which was used to determine the concentration of Lv. Our results indicated the visible-Fenton reaction is an easy, mild, green, efficient, and general approach to obtain GQDs; the FRET strategy for detection of Lv has high merit, not only for the wide linear test range (0.001–1500 ng/mL), lower limit of detection (LOD, 0.9 pg/mL), excellent sensitivity (26,407.8 CPS/(ng/mL)), selectivity, and reproducibility for Lv quantification, but also non-toxicity, safety for humans and the environment, easy-to-use, and time-saving. 

## 2. Materials and Methods

### 2.1. Materials and Characterization

GO was purchased from Shenzhen Turing Technology Co., Ltd., China. Hydrogen peroxide (H_2_O_2_, GR, 30 wt.% in H_2_O), ferric chloride hexahydrate (FeCl_3_·6H_2_O), ammonia aqueous (NH_3_·H_2_O, 25%), polyvinyl pyrrolidone (PVP), and ascorbic acid (AA) were purchased from Shanghai Aladdin Biochemical Technology Co. Ltd., Shanghai, China. 1-(3-dimethylaminopropyl)-3-ethylcarbodiimide hydrochloride (EDC) and N-hydroxysuccinimide (NHS) were purchased from Sigma-Aldrich. Ultrapure water with a resistivity of 18.2 MΩ prepared on a FLOM-FDY system was used throughout all experiments. 

A solar simulator (Shanghai Yanzheng Experimental Instrument Co., Ltd., China) composed of different colors of LEDs was employed as a catalysis light source with wavelength range in the range of 400–900 nm. The ultraviolet-visible (UV-Vis) absorption spectra was measured via a UH5300 spectrophotometer (Hitachi, Japan). A FluoroMax-4 (HORIBA Jobin Yvon, Edison, NJ, USA) fluorescence spectrometer was used to record the PL spectra. The crystalline structure was detected by an XRD (Bruker D8 ADVANCE, Bruker AXS, Germany) with Cu-K*α* radiation (λ = 1.5406 Å). The size and morphology of GQDs were observed by a H-7650 TEM (Japan). The high-resolution image of GQDs was observed with a field emission electron microscope (HRTEM, Tecnai G2 F30 Hillsboro, OR, USA). The surface morphology and thickness of the GQDs were characterized via an AFM (Seiko SPA400, Tokyo, Japan). Raman spectra were finished with a DXR Raman Microscope (Thermo Scientific, USA). FTIR spectra were obtained with a Nicolet IN10 FTIR spectrometer (Thermo Fisher Scientific, Waltham, MA, USA) with a resolution of 4 cm^−1^ from 4000 to 500 cm^−1^. An XPS (Thermo ESCALAB 250XI, America, with a radiation source Al K*α*-1486.6 eV) was used to analyze the relative content of carbon and oxygen in the samples. Zeta potential was measured by a Zetasizer Nano (Malvern, UK)

### 2.2. Fabrication of rGO

rGO was fabricated via reduction of GO with AA as a reducer and PVP a surfactant to prevent aggregation of rGO. At room temperature, 0.01 g GO was fully dispersed in 100 mL pure water with stirring for 15 min, the pH value of the solution is 3.4 and the color is dark brown. Dropping addition of 25% ammonia water into above solution till the pH of the solution is 9.5, then 100 mL 2% PVP (in weight) aqueous solution was mixed with the GO solution. After complete stirring, 5 mL 0.02 M AA aqueous were dropwisely added into the solution. Then the reaction solution was heated to 70 °C and kept constant temperature for 80 min. In the reduction process, the color of the solution gradually became darker and finally appeared dark black, indicating GO was reduced to rGO. After centrifuged at 12,000 rpm three times, the sample was dried in a thermoelectric thermostat drying box at 45 °C. The final rGO sample was preserved in a brown glass bottle for later use.

### 2.3. Preparation of GQDs

The visible-Fenton reaction was carried out in a solar simulator consisting of a series of LED cold light sources (wavelength 400–900 nm) for photo catalysis, without a circulating water setup for cooling the reaction tubes. Typically, 20 mL 1 mg/mL GO aqueous solution, 1 mL 1.0 × 10^−3^ M FeCl_3_ aqueous solution and 1 mL 30% hydrogen peroxide were mixed in a quartz tube under vigorous stirring. The pH value of the mixture was adjusted to 4. After reaction for 6 h, the product was centrifuged at 12,000 rpm, then filtered with a 0.22 μm microporous membrane. After dialysis in ultra-pure water for 24 h, small molecules such as iron, chloride ion, and a small amount of hydrogen peroxide were removed, a light-yellow solution of GQDs was obtained. The GQD powder was further prepared by removing water under evaporation at 50 °C. 

### 2.4. Fabrication of Amine Functionalized GQDs (afGQDs)

For easy conjugation of Anti-Lv-mAb and GQDs, GQDs was modified with ammonia solution. The fabrication process is similar to [54] with a slight change. 1 mg/mL aqueous GQDs was mixed with ammonia solution (25% in water) as volume ratio 1:1.1 at room temperature; the solution was heated up to 200 °C in a closed reactor, and kept constant temperature overnight then cooled at room temperature for 2 h; the solution was further heated up to 100 °C for 1.5 h to vaporize excess ammonia.

### 2.5. Production of Anti-Lv mAbs

Vtg and Lv of Japanese flounder (*Paralichthys olivaceus*) were purified and identified according to our previous studies [55,56]. Anti-Lv-mAbs were obtained by the standard procedures of cell fusion and hybridoma screening, and then purified by affinity chromatography [56].

### 2.6. Conjugation of afGQDs and Anti-Lv mAbs

The antibody activation and conjugation of Anti-Lv-mAbs and afGQDs were similar to Reference 54 with a small adjustment. 0.99 mL 4 μg/mL Anti-Lv mAbs was mixed with 100 μL 0.1 M MES solution with 0.4 mg EDC and 1.1 mg NHS companying a vigorous stirring for 15 min at 27 °C. The activated Anti-Lv-mAbs was incubated with afGQDs (10 μg/mL) at a volume ratio of 1:1 at 37 °C for 30 min. To remove the unconjugated molecules, the conjugate was washed five times with PBS buffer solution by ultracentrifugation. The final conjugate (Anti-Lv-mAbs/afGQDs) was used as fluorescent nanoprobe in the following experiments.

### 2.7. Optimized Amount of rGO

For the π–π stacking interaction, electrostatic attraction, and Van der Waals force, the Anti-Lv-mAbs/afGQDs can be attracted to the surfaces of rGO. When the distance between the donor (Anti-Lv-mAbs/afGQDs) and the acceptor (rGO) is less than the Förster radius (<10 nm), effective FRET can occur because rGO is a broad-spectrum absorbing material [53,54]. For a certain concentration of Anti-Lv-mAbs/GQDs, there is a best amount of rGO for highest PL quenching efficiency. 0.5 mL mAb-afGQDs was respectively added into 0.1 mL rGO aqueous solutions (0.5, 1.0, 1.5, 2.0, 2.5,3.0, 3.5, and 4.0 mg/mL respectively), after incubated for 30 min at room temperature, the PL spectra were measured. The best amount of rGO was determined by the highest PL quenching ratio.

### 2.8. Immunosensing for Lv

The optimal amount of rGO was added into the Anti-Lv-mAb/afGQDs solution and fully dispersed. For establish the standard testing curve, each 0.6 mL Anti-Lv-mAb/afGQDs/rGO solutions were respectively added into different concentrations of standard Lv aqueous solutions (0–1500.0 ng/mL), each total volume was 1 mL by addition PBS buffer solution. After incubation for 12 min at room temperature, the PL spectra of the mixture were measured, and the data were saved for later analysis. Each experiment was repeated three times.

### 2.9. Sensitivity, Selectivity and Reproducibility

The LOD was determined by *S*/*N* = 4 and the established standard calibration curve, in which *N* and *S* are the PL intensities of the blank and the sample. To evaluate the selectivity of the immunosensor, ovalbumin (OVA, derived from egg white), and bovine serum albumin (BSA) were used as interferences [56,57,58]. To verify the reproducibility of the immunosensor, Lv (1 ng/mL) was dispersed into city water for test. The experimental procedure is similar to Section 2.8. All the above experiments were performed three times.

## 3. Results

### 3.1. Properties of rGO

**Figure 1:**
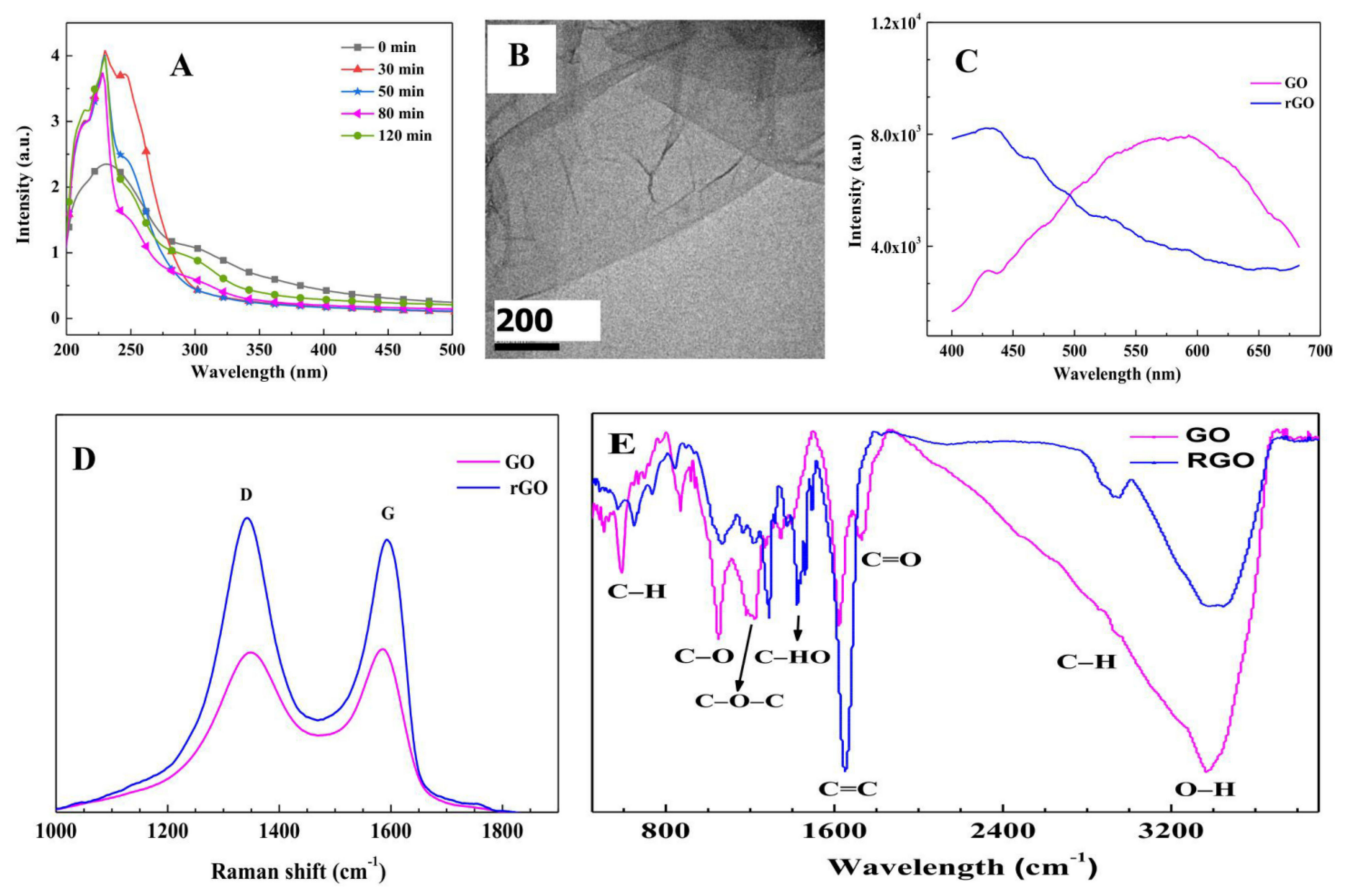
Characterization of graphene oxide (GO) and reduced graphene oxide (rGO). (**A**) UV-Vis absorption spectra at different reduction times when GO was reduced to rGO; (**B**) TEM image of rGO; (**C**) photoluminescent (PL), (**D**) Raman, and (**E**) FTIR spectra of GO and rGO.

During the process of GO being reduced to rGO, the intermediate product was sampled at different times (0, 30, 50, 80, and 120 min, respectively). After washing and centrifugation, the UV-Vis absorption spectra of these sampled products were measured. The results are shown in Figure 1A. GO has a broad absorption peak at 232 nm and a wide shoulder peak at 300 nm. Generally, the peak near 230 nm is attributed to the π→π* electronic transition of C=C within the aromatic sp^2^ domains, and the peak of 300 nm is assigned to the edge transition [59]. Both the *n*→π* transition of the surface groups and the π→π* charge transfer transition of the edge sp^2^ carbon domain contribute to this transition [59]. Within 80 min of reduction, a sharp peak at 228 nm became obvious and narrowed gradually; however, the peak at 300 nm decreased, indicating O in GO was gradually removed. Continuing the reduction till 120 min, the peak at 300 nm began to increase, implying the reduced product could be oxidized again under heating at 70 °C in air. Above results show ~80 min is an appropriate reduction time for GO changed into rGO. The size of rGO is within 0.5–6 μm (see Figure 1B). Comparing to GQDs, both of GO and rGO are weak fluorescent matters, the PL intensities of GO and rGO are only 1/80 of GQDs (see Figure 1C and Figure 6B). After reduction, the PL peak of GO from near 575 nm moves to 430 nm. 

Raman spectroscopy is often used to identify the intrinsic features of sp^2^ carbon (the ordered structural degree of the graphitization, which is the symbol of the Raman active E_2g_ mode and characterizes the crystalline nature of a carbon material) and disordered sp^3^ carbon (such as oxygen groups, heteroatom doping, defects, and size reduction) [59,60,61]. Two typical peaks, named the D band (1340.5/1349.4 cm^−1^) and the G band (1592.2/1585.6 cm^−1^) were observed for rGO/GO (Figure 1D). The intensity ratio of *I*_D_/*I*_G_ is strongly related to the sp^2^ cluster sizes and the average distance between the two defects [61]. For rGO and GO, *I*_D_/*I*_G_ were estimated to be 1.076 and 1.010 respectively. The ordered sp^2^-domain size (*L*_a_) can be estimated by the following relation [60,61],

(*I*_D_)/(*I*_G_) = (−126 + 0.033 *λ* [Å])/(*L*_a_[Å]) (1)

in which *λ* is the laser wavelength of Raman spectrometer (532 nm). The calculated *L*_a_s for rGO and GO are about 4.6 nm and 5.0 nm, respectively; the ordered size of rGO is slightly larger than that of GO. The main reason is that partial oxygen groups were removed from GO in the reduction process. 

FTIR spectroscopy was used to identify the functional groups before and after the reduction of GO (see Figure 1E). The FTIR spectrum of GO shows the presence of hydroxyl (–OH (3377 cm^−1^), carbonyl C=O (1729 cm^−1^), aromatic C=C (1630 cm^−1^), epoxy C–O–C (1224 cm^−1^), and C–H (872 cm^−1^ [62,63] and 592 cm^−1^ [62]). To prevent the aggregation of rGO, PVP was used as a surfactant agent. The peaks at 1427 cm^−1^ and 1296 cm^−1^ in the FTIR of rGO indicate the vibration of heterocyclic [64,65] in PVP, and the peaks at 845, 735 and 652 cm^−1^ are out of plane C—H bending, rocking and OH wagging in PVP [64,65]. Comparing to the GO, the C=C bond increases greatly. At the same time, the C=O, C–O, and C–O–C decrease obviously, implying partial oxide-containing groups were broken in the reduction process, which coincides well with the result of UV-Vis absorption and Raman experimental results. 

### 3.2. Morphology and Structure of GQDs

**Figure 2:**
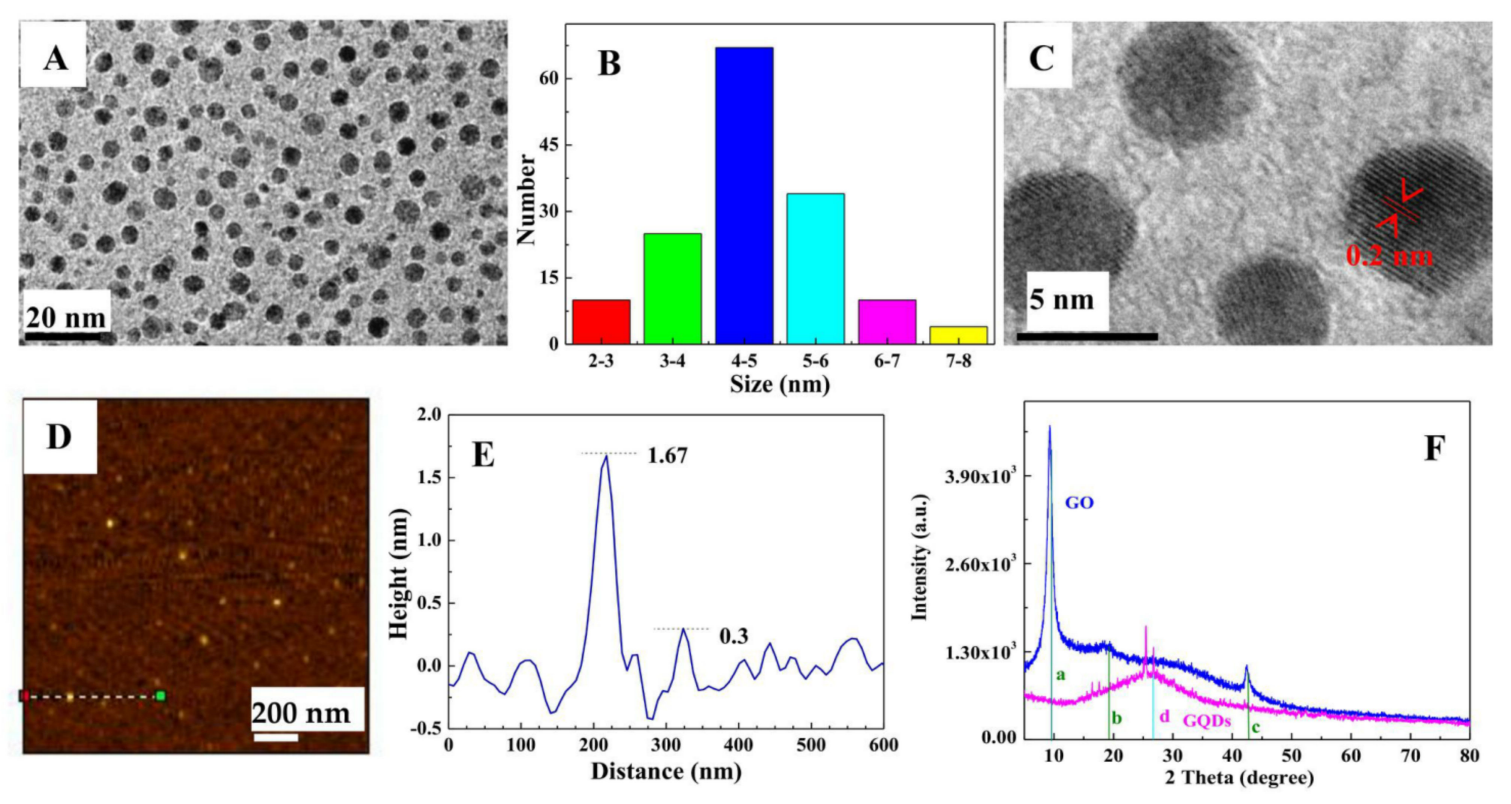
Morphology and structure of the graphene quantum dots (GQDs). (**A**) TEM, (**B**) size distribution, (**C**) HRTEM, (**D**) AFM, (**E**) the height profile corresponding to the line in (**D**), and (**F**) XRD patterns of GQDs and GO.

After the visible-Fenton reaction, the as-prepared GQDs have excellent dispersion in water and a narrow size distribution at 3–6 nm (Figure 2A,B); the HRTEM result indicated a regular crystalline structure with a lattice distance of 0.2 nm (Figure 2C and Figure S1), which is well-consistent with the published results [6,26,27]; the AFM pattern (Figure 2D,E) shows the height profile of GQDs is about 0.3–1.67 nm, corresponding to one to five layers of Gr. The above results coincide well with those of GQDs fabricated using the UV-Fenton method [26,27,28]. The XRD pattern of GO (blue curve in Figure 2E) shows three peaks at 9.56°, 19.18°, and 42.48°, which are the (001) and (002) planes of GO (PDF No. 00-065-1528), and the (100) plane of carbon (PDF No. 00-089-8498). The XRD pattern of GQDs (rose-red curve) shows a wide peak positioned at 26.6° is near to the (002) crystal face of exfoliated graphite [59], revealing the graphene structure of GQDs. According to the Debye–Scherrer equation, the calculated interplanar crystal spacing of GQDs is 0.365 nm, which is greater than that of graphite (0.335 nm) [59]. The main reason is that abundant oxygen-containing functional groups were formed on the lamellar surfaces or edges of the GQDs in the process of visible-Fenton reaction, resulting in GQDs possessing good water solubility.

### 3.3. Optical Properties of the GQDs 

**Figure 3:**
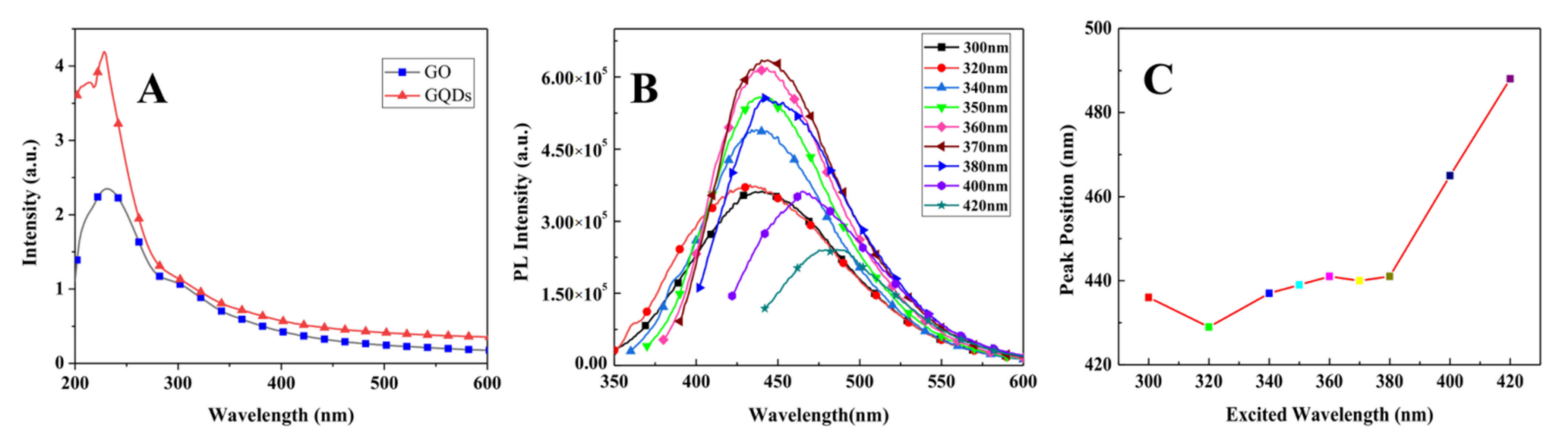
(**A**) UV-Vis absorption spectra of GQDs, GO is for comparison, (**B**) PL spectra of GQDs at different exciting wavelengths, and (**C**) PL peak positions changing with the excitation wavelengths.

Figure 3A shows the UV-Vis absorption spectra of GQDs and GO; GQDs show a sharp peak at 228 nm attributed to the π→π* electronic transition of C=C within the aromatic sp^2^ domains (e.g., core state) [59,60] and a shoulder peak at 300 nm assigned to the edge transition [61]; a long absorption tail extending into the visible range is similar to the characteristic absorption of semiconductor materials, implying that GQDs resemble a semiconductor. Many reports show that the PL spectra of GQDs are excitation-dependent [19,20,21,24,25,26,60] because of the presence of multi-emissive centers [60]. For the as-prepared GQDs, under excitation at 300, 320, 340, 350, 360, 370, 380, 400, and 420 nm, the PL peaks are at 436, 429, 437, 439, 441, 440, 441, 465, and 488 nm, respectively (Figure 3B); the peak positions of the PL spectra are almost invariant (~440 nm) within 300–380 nm excitation, showing an excitation-independent property (Figure 3C); the emissive intensities increase with the excitation wavelength in the range of 300–370 nm, with a maximum under 370 nm excitation, the quantum yield is up to 27.8%; while excitation in the range of 400–420 nm, the PL peak positions indicated a quick red-shift, showing a strong excitation-dependent property (Figure 3C). The excitation-independent PL peaks are regarded as the carbon core (sp^2^ domain) of the GQDs [60], and the excitation-dependent PL peaks are related to the transitions of the surface states originating from the hybridization between the attached groups and the carbon core [60]. For emitting bright blue fluorescence, the GQDs are excellent candidates for fluorescent sensors. 

The controlled experiment was carried out under dark conditions with Fe^3+^/H_2_O_2_ or with visible light irradiation and a lack of a Fenton agent. the colors of GO aqueous solutions did not show obvious change within several hours, indicating that light irradiation and Fe^3+^/H_2_O_2_ are the necessary conditions for the Vis-Fenton reaction of GO, which are similar to the UV-Fenton result [26].

### 3.4. Surface Chemistry of the GQDs

**Figure 4:**
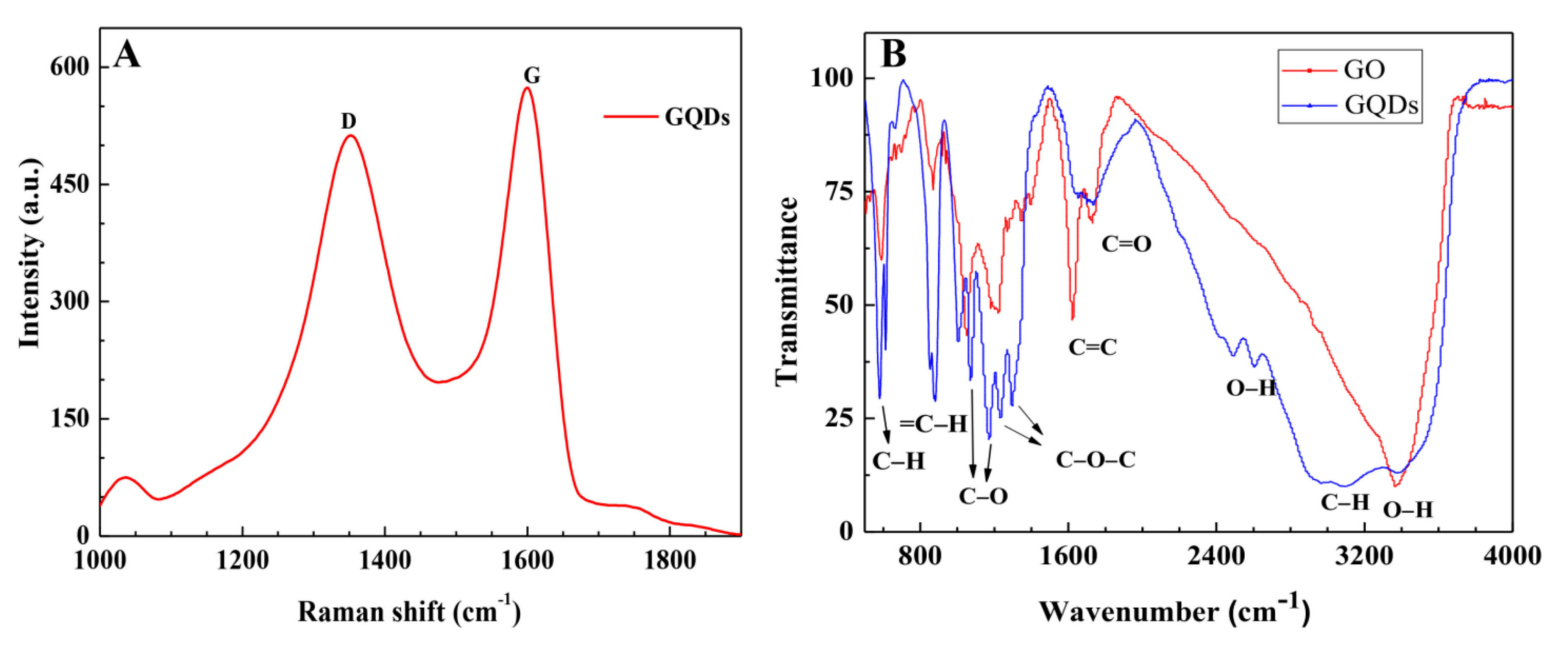
(**A**) Raman and (**B**) FTIR spectra of the GQDs. In (**B**), GO is for comparison.

The D band (1354 cm^−1^) and the G band (1600 cm^−1^) were observed in the Raman spectra of GQDs (Figure 4A). The intensity ratio of *I*_G_/*I*_D_ was estimated to be 1.116. The ordered sp^2^-domain size (*L*_a_) of the GQDs was estimated by equation (1) to be 5.5 nm, which is slightly larger than that of the GO. The main reason is that when a micro-sized GO is broken into nano-sized pieces, the breaking is easily initiated at the defects on the GO [26,27,28]; and in the reaction proceeding, the structures of these small pieces become more ordered, and oxygen groups mainly positioned at the edges of these small sheets, which resulted in the ordered size of GQDs being slightly larger than that of GO. 

**Figure 5:**
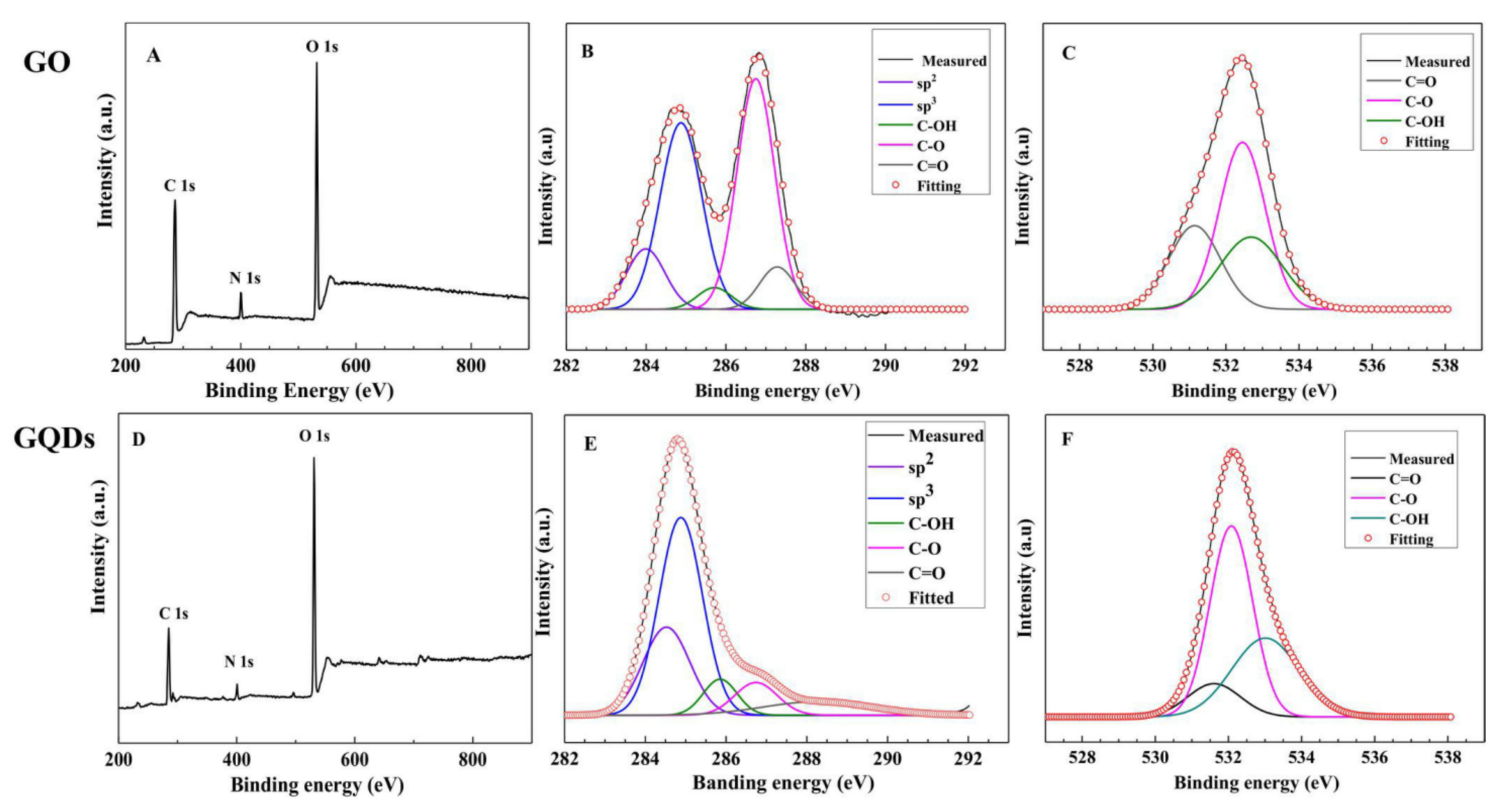
XPS spectra of GO (upper) and GQDs (lower). (**A**,**D**): survey scan spectra; (**B**,**E**): high resolution spectra of C1s; (**C**,**F**): high resolution spectra of O1s.

The surface chemistry of the GO and GQDs was characterized using FTIR (Figure 4B) and XPS (Figure 5). The FTIR of GQDs presents the C=C benzene ring (1621 cm^−1^); the carbonyl group C=O (1720 cm^−1^) [66]; C–O at 1071 cm^−1^ and 1170 cm^−1^ [66,67,68], C–O–C vibration in epoxy (1232 cm^−1^ and 1293 cm^−1^) [69], and O–H of COOH at 2490 cm^−1^ and 2605 cm^−1^ [70]; C–H vibration at 2970 cm^−1^ [71]; the broad peak at 3372 cm^−1^ is attributed to the bending vibration of hydroxyl OH [66,72]; and the peaks at 586 cm^−1^ and 880 cm^−1^ are C–H and =C–H, respectively [62]. In comparison with GO, the FTIR of GQDs shows the intensification of C=C, C–O–C, and OH, implying that the crystalline structure of GQDs is more ordered than that of GO, and also coinciding well with the result of Raman (Figure 4A). GQDs and GO possess rich carboxylic, hydroxyl, epoxy, and carbonyl groups, resulting in good hydrophilicity. 

The XPS survey scan of GO/GQDs (Figure 5A,D) exhibits three peaks at 286/285, 400/402, and 532/533 eV, indicating the existence of C1s, N1s, and O1s; the very weak N1s that appeared in the XPS of GQDs (Figure 5D) originated from GO (Figure 5A). The deconvolution of the high resolution C1s XPS spectra of GO (Figure 5B) and GQDs (Figure 5E) reveals five peaks, confirming the presence of sp^2^ C (C=C, 283.99 eV), sp^3^ C (284.88 eV), C–OH (285.71 eV), C–O–C (286.73 eV), and C=O (287.28 eV); the three decompositions of O1s for GO (Figure 5C) and GQDs (Figure 5F) confirm the C=O (531.14 eV), C–O (532.45 eV), and C–OH (532.68 eV) presence in the GO and GQDs. When comparing the high-resolution XPS spectra of C1s and O1s for GO and GQDs, the relative intensity of sp^2^ C increases greatly via the visible-Fenton reaction, indicating that the GQDs have a more ordered graphite structure of sp^2^ C and also coinciding well with the results of the HRTEM (Figure 2C), Raman (Figure 4A) and FTIR (Figure 4B); the relative ratios of C–O–C and C=O bonds in GQDs decreased after the visible-Fenton reaction, partially because of epoxy and alkoxy bonds breaking in the reaction process. At the same time, the intensity of the C–OH bond increases, meaning that more COOH groups were produced during the visible-Fenton reaction. The main reason might be that C=C and C–C bonds in GO were broken, leading to more periphery carbons being oxidized into the carboxylic (COOH) groups, which is benefit of GQDs possessing good water solubility, bio-compatibility, and easy amine modification. Except for C, O, and N, the small peaks near to 234, 496, and 555 eV are the Auger peaks of Mg and Na; and the weak peak near to 717 eV is the Fe 2p. Mg and Na originated from GO. FeCl_3_ was a catalyst in the visible-Fenton reaction via dialysis. Fe was not fully removed, so Fe was mainly derived from the catalyst. GQDs dispersed in water indicated a strong negative Zeta potential of −25.0 mV (Figure S2), which also showed that GQDs have good stability and hydrophilicity.

### 3.5. Optical Properties of afGQDs and Anti-Lv-mAb/afGQDs (Conjugate)

**Figure 6:**
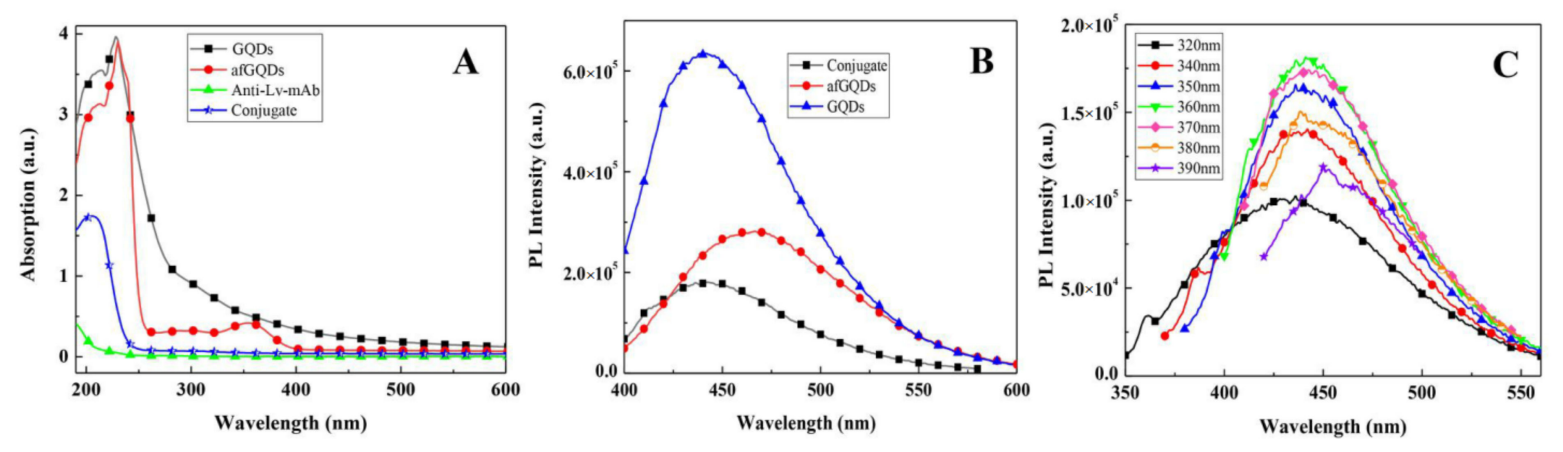
(**A**) UV-Vis absorption spectra of afGQDs, Anti-Lv-mAb and the conjugate of Anti-Lv-mAb/afGQDs; (**B**) PL spectra of afGQDs and Anti-Lv-mAb/afGQDs under excitation at 370 nm; and (**C**) PL spectra of Anti-Lv-mAb/afGQDs under various excitation wavelengths. In (**A**,**B**), GQDs is used as a reference.

The FTIR and XPS results showed that the –COOH and C–O–C presence in GQDs, under heating, can cause two reactions by nucleophilic interaction between C–O–C and NH_3_ and condensation (reaction) between –COOH and NH_3_, so the GQDs were easily amino-modified [73,74]. The amine functionalized GQDs were characterized by their UV-Vis absorption spectra, Zeta potential, and PL spectra. Similar to GQDs, afGQDs indicated a strong absorption at 230 nm corresponding to π→π * electronic transition of C=C within the aromatic sp^2^ domains. Another peak at 360 nm should be related to –NH_2_ and oxygen-containing groups in afGQDs (see Figure 6A) [60]. The zeta potential for afGQDs is 10.5 eV (Figure S2). Compared to the GQDs, the afGQDs indicate a positive shift in zeta potential, also implying that the amine groups are ultimately incorporated into the GQDs. Compared to the GQDs, the PL intensities of the afGQDs decreased (Figure 6B, 370 nm excitation); the fluorescence peak of afGQDs was red-shifted to ~ 468 nm, which might be due to the more defect states after amine modification. The successful amine modification of GQDs is a benefit of that conjugation of anti-Lv-mAb and afGQDs.

The conjugation of Anti-Lv-mAb and afGQDs was probed via the UV-Vis absorption (Figure 6A) and PL spectra (Figure 6B,C). For the Anti-Lv-mAb, a very low absorption peak near 200 nm was observed; the conjugate showed an absorption peak at 210 nm. The intensity was much higher than that of the Anti-Lv-mAb but lower than that of afGQDs. After the Anti-Lv-mAb coupled with the afGQDs, the absorption peaked at 360 nm related to –NH_2_ in afGQDs is while invisible in the conjugate, implying that –NH_2_ in afGQDs reacted with –COOH in the Anti-Lv-mAb, so the Anti-Lv-mAb conjugated with the afGQDs successfully. The peak position of the conjugate blue moved to 442 nm (see Figure 6B), should be –NH_2_ in afGQDs reacted with –COOH in Anti-Lv-mAb to form –CONHR, and partial defect state emission from afGQDs changed into an intrinsic state emission [75].

The PL spectra of the conjugate are excitation-independent within 340–380 nm of excitation (Figure 6C), similar to the GQDs (Figure 3B); the maximum PL was obtained under excitation at 360 nm. Thus this wavelength was determined as the exciting wavelength for the below experiments.

### 3.6. PL Quenching (“OFF”) between rGO and Anti-Lv-mAb/afGQDs (Conjugate)

**Figure 7:**
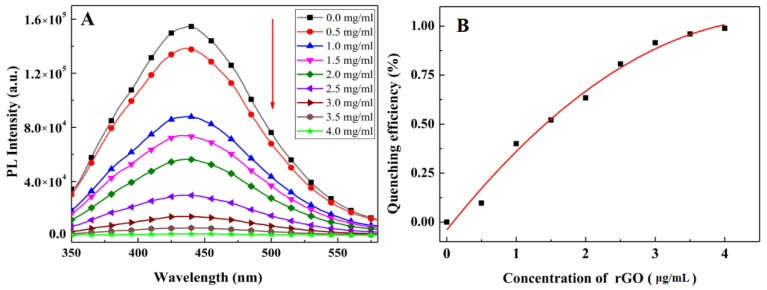
PL quenching of Anti-Lv-mAb/afGQDs after addition of different amounts of rGO. (**A**) PL spectra, and (**B**) quenching efficiencies vary with the concentrations of rGO.

Upon addition of rGO to the conjugate (Anti-Lv-mAb/afGQDs), FRET could be observed for energy non-radioactively transferred from the conjugate to the rGO. The PL quenching depends on the concentration of rGO (Figure 7A). The quenching efficiency ((PL intensity without rGO–PL intensity with rGO)/PL intensity without rGO) increased with the amount of rGO. With the addition of 4.0 μg/mL rGO to the Anti-Lv-mAb/afGQD solution, the quenching efficiency was almost 1 (Figure 7B), indicating the entire PL originating from the conjugate was quenched by rGO; thus, 4.0 μg/mL rGO was selected as the best amount for Lv detection.

### 3.7. PL Recovering (“ON”) for Detection of Target Antigen (Lv)

**Figure 8:**
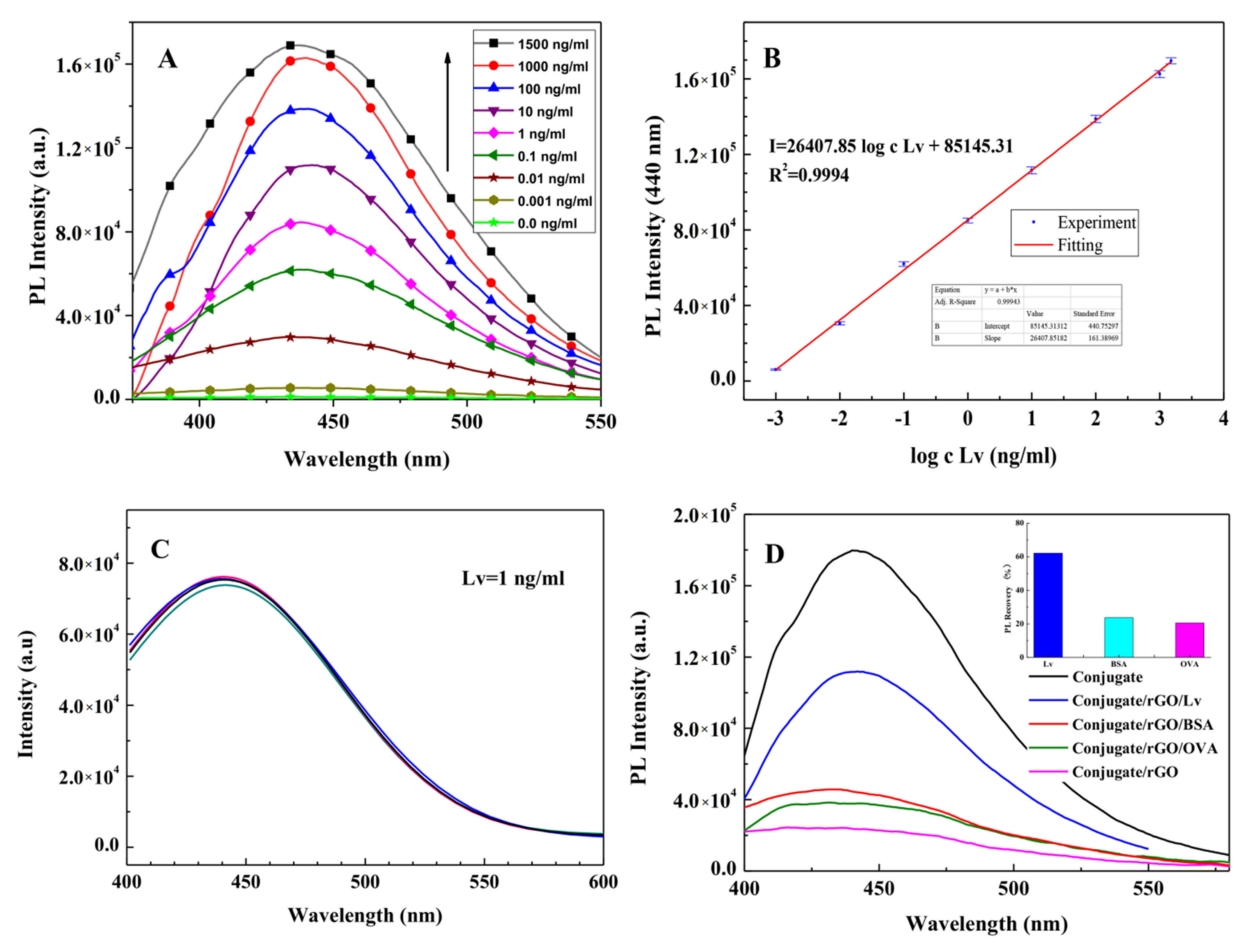
(**A**) PL “ON” after addition of different concentrations of Lv (0, 0.001, 0.01, 0.1, 1.0, 10, 100, 1000, and 1500 ng/mL) into Anti-Lv-mAb/afGQDs/rGO solutions; (**B**) the linear relationship between the PL regaining intensity with the logarithm concentrations of Lv from 1 pg/mL to 1500 ng/mL; (**C**) reproducibility, and (**D**) immunosensing behavior against specific (Lv) and non-specific antigen (BSA and OVA). In (**C**), addition of same amount of Lv (1 ng/mL) into 5 parallel samples of Anti-Lv-mAb/afGQDs/rGO solutions; and in (**D**), the concentration of Lv is 10 ng/mL, and OVA and BSA are 1.5 μg/mL.

Upon the addition of the target antigen, Lv, into the Anti-Lv-mAb/afGQDs/rGO (conjugate/rGO) solution, the strong and specific immune reaction between Lv and Anti-Lv-mAb impelled rGO escaping from the complex of Anti-Lv-mAb/afGQDs/rGO, the PL of the conjugate was regained. With the subsequent increase in Lv, due to a large number of rGO departing from the Anti-Lv-mAb/afGQDs/rGO complex, resulting in the PL intensity enhancing (Figure 8A). The linear relationship between PL intensities (*I*) and logarithm of Lv concentrations (log *c* Lv) was shown in Figure 8B (calibration graph). The mathematical expression can be written as, *I* = 26,407.85 log *c* Lv + 85,145.31 with a regression coefficient (*R*^2^) of 0.9994. The LOD for Lv was found to be 0.9 pg/mL (*S*/*N* = 4), the linear detection range of 1.0 pg/mL–1500 ng/mL, and the sensitivity of 26,407.8 CPS/(ng/mL). Comparing to the current detection methods (Table 1), the PL “ON-OFF” technique in this work has a wide linear detection range, the almost lowest LOD, and high sensitivity. The detection time is 12 min, which is lower than that reported earlier [42].

### 3.8. Reproducibility, Selectivity and Stability of the Immunosensor

Reproducibility, selectivity, and stability of the immunosensor are shown in Figure 8C,D. After the addition of the same amount of Lv (1 ng/mL) into 5 parallel samples of Anti-Lv-mAb/afGQDs/rGO solutions and incubating for 12 min at room temperature, the PL spectra of the mixtures were measured (Figure 8C). The relative standard error was calculated to be about 1.20%, indicating good repeatability of the immunosensor. The selectivity of the immunosensor was tested by using BSA and OVA as interferences, the concentrations of BSA and OVA (1.5 μg/mL) are 150 times of the Lv (10 ng/mL), the fluorescence regaining results were presented in Figure 8D. The PL regaining ratios of Lv, BSA, and OVA are 62.13%, 23.70%, and 20.51%, respectively (see the inset in Figure 8D). The PL regaining ratio for Lv is much higher than that for BSA and OVA, implying that the “ON-OFF” immunosensor has good selectivity for Lv. The accuracy of the present FRET method was determined by testing the Lv recovery ratios in three city waters (1 ng/mL). Based on the PL spectra and the linear relationship between PL intensities (*I*) and logarithm of Lv concentrations (log *c* Lv) in Figure 8B, the measured recovery ratios are 95.73%, 99.48%, and 101.13%. The relative error is within 4.5%, indicating good reliability of the present FRET method. 

The afGQD was found to be very stable at room temperature for several months. Within one month and at 4 °C, the PL intensity of the Anti-Lv-mAb/afGQDs conjugate decreased slightly (5%), mainly due to the Anti-Lv-mAb. For good stability, the Anti-Lv-mAb and afGQDs can be preserved separately at 4 °C, then mixed at the beginning of the experiment. The response time of the sensor is very short (~10 s), and the total detection time for Lv from the label-free FRET immunosensor is only 12 min, which is the most efficient and rapid detection of Lv. 

## 4. Conclusions

In summary, rGO was obtained by reduction of GO, and bright blue fluorescent GQDs were prepared using visible-Fenton catalysis reaction with GO as a precursor. The morphology, structure, surface chemistry, and photo-physical properties of rGO and GQDs were well characterized. Based on the FRET between afGQDs conjugated Anti-Lv-mAb and rGO, the fluorescent “ON-OFF” label-free immunosensing for detection of Lv of Paralichthys olivaceus was constructed. The immunosensor indicated a wide linear test range (0.001–1500 ng/mL), with very low detection limit (0.9 pg/mL), excellent sensitivity (26,407.8 CPS/(ng/mL)), selectivity, reproducibility, and quick speed for Lv sensing. This optical label-free immunosensor has promising application in environmental estrogen biomarker tests.

## Figures and Tables

**Table 1:** Comparison the LODs and test ranges for various immunosensing methods for Vtg/Lv detection.

No	Sensors Type	Antibody Type	LOD (ng/mL)	Linear Range (ng/mL)	Reference
1	Electrochemical impedance sensor	mAb	42	1000.0–8000.0	[46]
2	Amperometric sensor	mAb	0.09	0.25–7.8	[47]
3	Optical surface plasmon resonance	mAb	1000	1000–10,000	[48]
5	Optical waveguide lightmode spectroscopy	polyAb	300(direct) 0.07(indirect)	600–12,000 ^a^ 3–100 ^b^	[51]
6	Surface-enhanced Raman scattering	polyAb	0.005	<0.2 ^c^	[49]
7	Optical waveguide lightmode spectroscopy	polyAb	Data not show	100–10,000 ^b^ 0.5–50 ^c^	[50]
8	ELISA ^d^	polyAb	Data not show	7.8–250	[55]
9	ELISA ^d^	mAb	0.75	1.95–250	[56]
10	FRET sensor	mAb	0.009	0.001–1500	This study

^a^: Direct manner, antibody was immobilized on the sensor surface. ^b^: Indirect manner, competitive measurement, Lv was applied for the immobilization. ^c^: Data not shown, judging from the result. ^d^: Sandwich enzyme-linked immunosorbent assay. mAb = Monoclonal antibody; pAb = Polyclonal antibody.

## Data Availability

The data used to support the findings of this investigation are available from the corresponding author upon request.

## Supplementary Materials

The following supporting information can be downloaded at: https://www.mdpi.com/article/10.3390/bios12040246/s1, Figure S1: The original HRTEM of the GQDs.; Figure S2: Zeta potential of GQDs and afGQDs.
