# Supplementary material for: Preparation of Graphene Quantum Dots by Visible-Fenton Reaction and Ultrasensitive Label-Free Immunosensor for Detecting Lipovitellin of Paralichthys Olivaceus
Source: Biosensors (Basel). 2022 Apr 15;12(4):246. doi: 10.3390/bios12040246 (PMC9024531; doi:10.3390/bios12040246)
Supplement: Supplementary file 1 [file biosensors-12-00246-s001.zip › biosensors-1525570-supplementary.pdf]

biosensors

Supporting information

Preparation of Graphene Quantum Dots by Visible-Fenton Reaction and Ultrasensitive Label-Free Immunosensor for Detecting Lipovitellin of Paralichthys Olivaceus

Ailing Yang [1,*], Yue Su [1], Zhenzhong Zhang [2], Huaidong Wang [1], Chong Qi [1], Shaoguo Ru [2] and Jun Wang [2,*]

1 College of Physics & Optoelectronic Engineering, Ocean University of China, Qingdao 266100, China; 21170211095@stu.ouc.edu.cn (Y.S.); wanghuaidong@stu.ouc.edu.cn (H.W.); qichong@stu.ouc.edu.cn (C.Q.)
2 College of Marine Life Sciences, Ocean University of China, Qingdao 266003, China; zhangzhenzhong5391@stu.ouc.edu.cn (Z.Z.); rusg@ouc.edu.cn (S.R.)
* Correspondence: ailingy@ouc.edu.cn (A.Y.); wangjun@ouc.edu.cn (J.W.); Tel.: +086-532-66781204 (A.Y.)

Citation: Yang, A.; Su, Y.; Zhang, Z.; Wang, H.; Qi, C.; Ru, S.; Wang, J. Preparation of Graphene Quantum Dots by Visible-Fenton Reaction and Ultrasensitive Label-Free Immunosensor for Detecting Lipovitellin of Paralichthys Olivaceus. *Biosensors* **2022**, *12*, 246. https://doi.org/10.3390/bios12040246

Received: 10 December 2021
Accepted: 22 January 2022
Published: 15 April 2022

Publisher's Note: MDPI stays neutral with regard to jurisdictional claims in published maps and institu-tional affiliations.

Copyright: © 2022 by the authors. Submitted for possible open access publication under the terms and conditions of the Creative Commons Attribution (CC BY) license (https://creativecommons.org/licenses/by/4.0/).

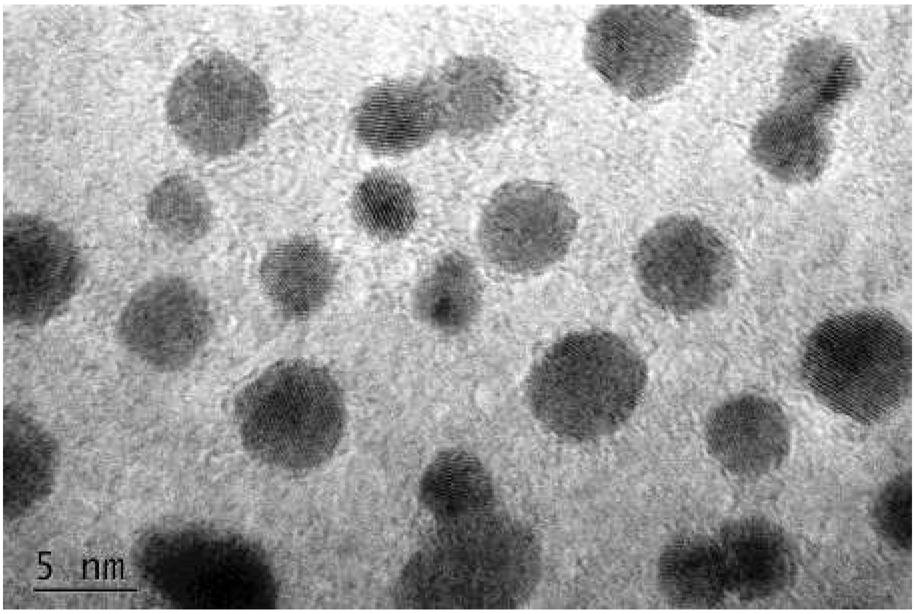

Figure S1. The original HRTEM of the GQDs

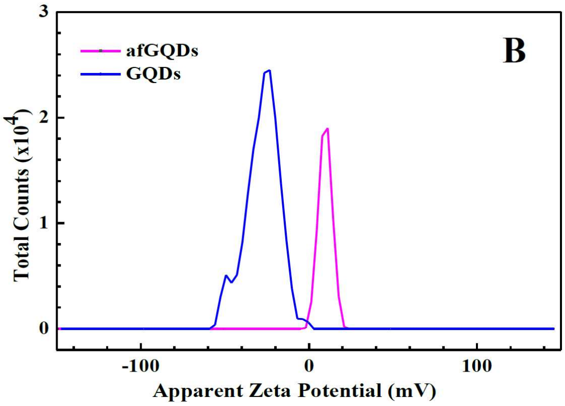

Figure S2. Zeta potentials of GQDs and afGQDs.
